# Comparative study of the McGrath™ videolaryngoscope blades and conventional laryngoscopy efficacy during mechanical chest compressions: Insights from a randomized trial with 90 anesthesiologists on objective and subjective parameters

**DOI:** 10.1371/journal.pone.0310796

**Published:** 2024-09-20

**Authors:** Tobias Golditz, Joachim Schmidt, Torsten Birkholz, Anja Danzl, Andreas Moritz, Andreas Ackermann, Andrea Irouschek

**Affiliations:** Faculty of Medicine, Department of Anesthesiology, University Hospital Erlangen, Friedrich-Alexander-Universität Erlangen-Nürnberg, Erlangen, Germany; CHU Nantes, FRANCE

## Abstract

**Aims:**

This study aimed to compare the efficacy and utility of the McGrath™ videolaryngoscope, using the Macintosh-like McGrath™ MAC blade and the hyperangulated McGrath™ MAC Xblade with a conventional Macintosh blade under simulated resuscitation conditions.

**Methods:**

A prospective, randomized study under conditions mimicking ongoing chest compressions was conducted with 90 anesthesiologists. Intubation success rates, time-to-vocal cords, time-to-intubate, and time-to-ventilate were measured. Additionally, the study assessed the subjective ratings and the perceived workload using the ‘NASA-task-load-index’ during the procedure.

**Results:**

The overall intubation success rate was device dependent 99–100%. The McGrath™ MAC and McGrath™ MAC Xblade showed faster visualization times compared to conventional blades. The MAC blade demonstrated superior performance in time-to-intubate and time-to-ventilate compared to both conventional and MAC Xblades. Despite excellent visualization, the MAC Xblade posed challenges in tube placement, reflected in a prolonged intubation time of >120 seconds in one case. Both MAC and MAC Xblade reduced potential dental injuries and interruptions to chest compressions compared to conventional laryngoscopes. User experience significantly impacted intubation times with conventional laryngoscopes, but this effect was mitigated with videolaryngoscopy. Participants reported lower stress and effort when using videolaryngoscopes, with the MAC blade rated superior in perceived time pressure.

**Conclusion:**

The study supports the superiority of videolaryngoscopy with a Macintosh-like blade over conventional laryngoscopy during mechanical chest compressions, particularly for less experienced users. The McGrath™ MAC blade, in particular, offers advantages in intubation time, user-friendliness, and reduced stress. However, the MAC Xblade’s challenges during tube placement highlight the need for further clinical validation. Continued research is essential to refine guidelines and improve resuscitation outcomes.

## Introduction

Endotracheal intubation in emergency situations is significantly more challenging than in controlled settings such as the operating room. This disparity is well documented, with evidence suggesting increased difficulty in airway management and a higher incidence of complications during emergency intubations [1]. In the prehospital setting, the challenges are compounded by a variety of factors, including adverse environmental conditions, suboptimal patient positioning, inadequate lighting, and human factors, including varying levels of provider expertise and fatigue. Together, these elements contribute to the complexity of prehospital intubations and often result in lower first-pass success rates. Studies have demonstrated markedly lower first-pass success rates for endotracheal intubation in the prehospital setting, ranging from 57% for paramedics [2] to 67% for non-physicians and up to 87% for physicians [3].

Recent advances have highlighted the potential advantages of videolaryngoscopy (VL) over direct laryngoscopy in improving intubation outcomes. VL has been associated with fewer esophageal intubations [4,5], reduced force application [6], better visualization of the vocal cords [7], shorter intubation times, and fewer interruptions during chest compressions [8]. A recent multicenter study has shown an advantage of videolaryngoscopy in critically ill patients [9].

An important distinction within VL devices is between traditional Macintosh-like blades and hyperangulated blades. The Macintosh-like blades provide a familiar technique for practitioners accustomed to direct laryngoscopy, while hyperangulated blades provide an optimized angle for glottic visualization. The use of hyperangulated blades offer specific advantages such as improved glottic visualization under difficult intubation conditions [10,11]. However, placement of the endotracheal tube (ETT) with these blades can be particularly challenging [11]. Especially ongoing chest compressions may exacerbate the difficulty due to movements of the patient.

Airway management during cardiopulmonary resuscitation (CPR) presents unique challenges and must be performed under significant time constraints and with the goal of minimizing interruptions in chest compressions. Traditionally, the choice of laryngoscope blade has depended on individual risk assessment and personal preference of the person providing the intubation. Airway management during resuscitation is often performed on the floor, further complicated by continuous chest compressions. Optimal positioning of both patient and intubator can often not be achieved or only to a limited extent. Due to the described difficulties of endotracheal intubation during resuscitation, its use is only recommended for well-trained teams and in situations where the chances of successful intubation are considered high [12–14].

In Parts of Germany, the McGrath™ series of videolaryngoscopes has been widely adopted by pre-hospital emergency services. The McGrath™ videolaryngoscope (Aircraft Medical Ltd., Edinburgh, UK) is particularly advantageous in the prehospital setting due to its portable, compact design, a bright LCD display, and interchangeable blades, including hyper-angulated blades (McGrath™ MAC Xblade) designed for difficult airways. To date, to the best of our knowledge there has been no direct comparison between a conventional laryngoscope and McGrath™ videolaryngoscope using a Macintosh-like blade and the hyperangulated X-blade during mechanical chest compressions in a typical resuscitation setting. Most probably, this lack of scientific data may underscore the importance of understanding the efficacy of different blades in this specific but essential scenario.

The study evaluates the effectiveness of conventional intubation versus the use of a Macintosh-like blades versus hyper-angulated blades in the McGrath™ videolaryngoscope in 90 anesthesiologists during mechanical chest compressions. A subanalysis investigates on the influence of the level of experience on the performance with each blade. By comparing these blades, we aim to gain insight into which type facilitates better intubation and patients’ safety.

## Materials and methods

### Study design and setting

The crossover manikin study presented here received approval from the local institutional ethics committee (Ethics Committee of the Friedrich-Alexander University Erlangen-Nürnberg; Reference number: 408_18 B). Prior to participation, all 90 individuals provided written and informed consent. Participants were recruited from Aug 25^th^ 2023 until Nov 16^th^ 2023. Participant data were anonymized, and access to information regarding individual performances was restricted to the research team exclusively.

The study was performed on an adult resuscitation manikin (Resusci Anne Advanced SkillTrainer, Laerdal Medical, Puchheim, Germany). The manikin was placed on the floor in a supine position. In order to reduce the mouth opening and to make the intubation conditions more realistic, a semi-rigid neck collar (Stiffneck^®^ Extrication Collar, Laerdal Medical, Puchheim, Germany) was fitted on the manikin. During intubation, mechanical chest compression was performed using LUCAS^®^ chest compression system (LUCAS 1 Chest Compression System, Stryker Medical, Portage, USA). During the intubation attempt, each participant was free to pause the mechanical chest compressions for a short time if necessary. The duration of the compression pause was documented.

Each participant performed endotracheal intubation with the McGrath™ MAC Videolaryngoscope (McGrath™ MAC Videolaryngoscope, Medtronic, Minneapolis, USA) using a single-use Macintosh-shaped McGrath™ MAC Blade Size 3 (MAC) and a hyperangulated single-use McGrath™ MAC Xblade (MX) as well as a conventional Macintosh laryngoscope blade size 3 (Rüsch® Polaris Single-Use Laryngoscope Blade MC 3, Teleflex Medical Ltd, Athlone, Ireland) (CON) in combination with a Heine F.O. SLIM LED metallic laryngoscope handle (Heine Optotechnik GmbH & Co. KG, Gilching, Germany). The order of the devices for intubation was randomized for each participant. Blades were used equally often as first, second and third device. The randomization was carried out using Research Randomizer (Urbaniak, G. C., & Plous, S. (2013). Research Randomizer (Version 4.0) [Computer software]. Retrieved on Aug 20, 2023, from http://www.randomizer.org).

**Fig 1:**
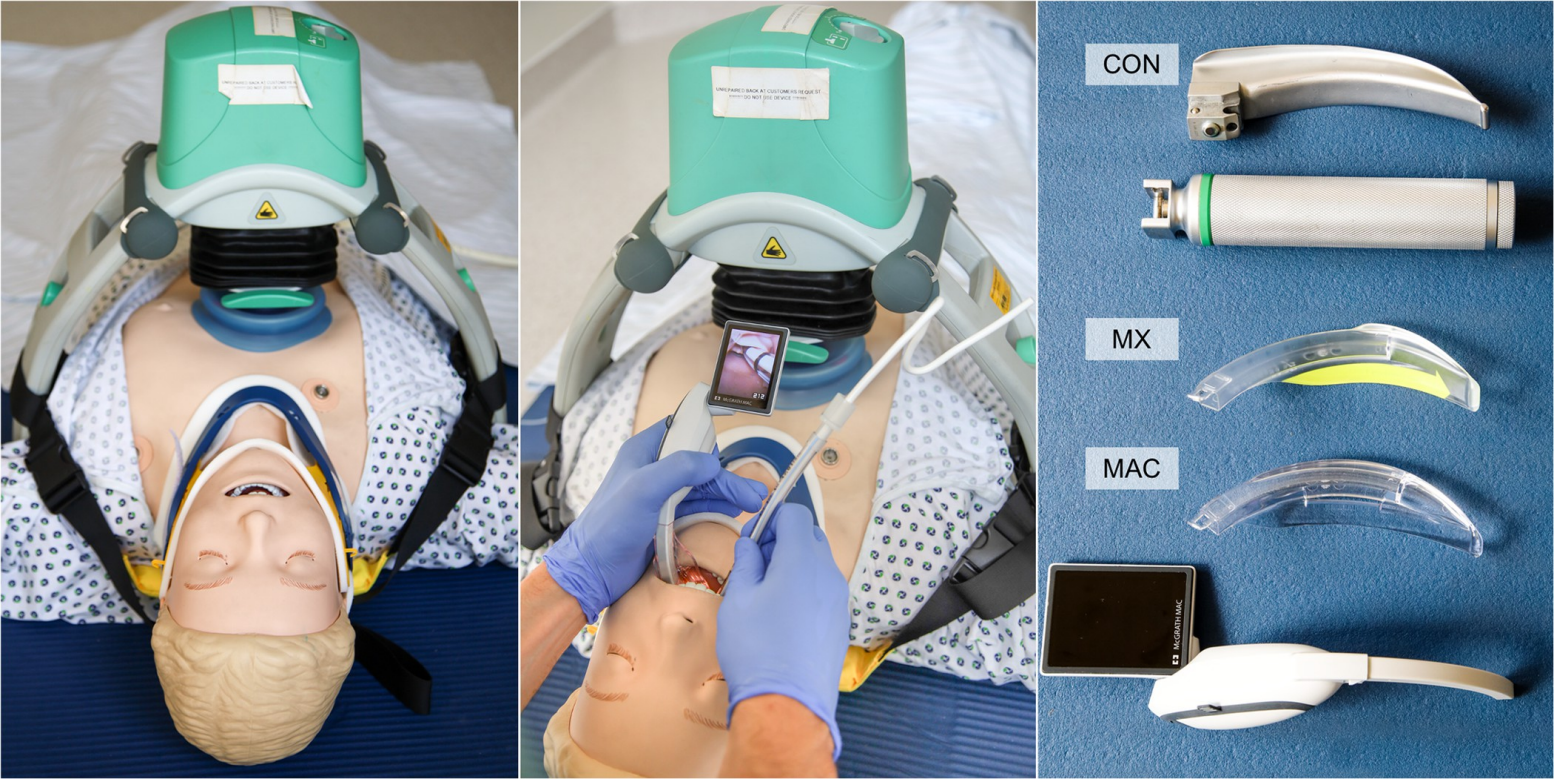
The figure shows an example of the study setup. The manikin is positioned supine on the floor. The ETI is performed by the participants under mechanical chest compressions (here with MAC as an example). The third image shows the devices used to secure the airway. The different blades are marked.

All intubations were performed with a 7.0mm cuffed Rüsch® ETT (Rüsch® Super Saftey Clear Tube, Teleflex Medical Ltd, Athlone, Ireland). A reusable 5.6mm outer diameter Rüsch® intubation stylet (Teleflex Medical Ltd, Athlone, Ireland) was used for each intubation. The participants formed the stylet individually prior to each intubation. For a better visualization, Fig 1 displays exemplarily the study setup.

### Participant population

The study included anesthesiologists from the Department of Anesthesiology at the University Hospital Erlangen, which is a tertiary care center conducting over 30,000 anesthesia procedures annually. Characteristics of the participants, including the level of experience, the frequency of intubations in adults, as well as the number of VL intubations, were recorded. Data were extracted from the electronic patient data management system (NarkoData, IMESO, Hüttenberg, Germany) for precise documentation. *Only the intubations performed during the induction of anesthesia on the patient in the operating room are recorded*. *As part of their anesthesiology training*, *the participants also take part in training courses on airway management*, *videolaryngoscopy and intubation on various difficult airway simulators*. Results are presented as median with interquartile range (IQR).

### Measurement

#### Objective assessment

Intubation time was meticulously measured for each attempt and device. To facilitate comparison across different devices, we established three distinct time points:

1. The "time-to-vocal cords" referred to the duration from the insertion of the blade between the teeth until the vocal cords were visualized.
2. The "time-to-intubate" indicated the time from blade insertion between the teeth until the ETT was deemed correctly positioned by each participant.
3. The "time-to-ventilate" represented the time from blade insertion between the teeth until the ETT was connected to a self-inflating resuscitation bag, and lung inflation was confirmed.

The primary endpoint focused on "time-to-intubate." Instances of esophageal intubations, attempts exceeding 120 seconds, or more than two intubation attempts (complete withdrawal of the device from the mouth and repositioning) were categorized as intubation failures. If an esophageal intubation went undetected, the intubation attempt ceased with no further attempt. Esophageal intubations were excluded from the statistical analysis of intubation times due to the absence of recorded "time-to-intubate" or "time-to-ventilate”.

To mitigate potential interobserver bias, stopwatch measurements were conducted by a solitary member of the research team. We documented the success rate of intubations, the number of intubation attempts, optimization maneuvers (such as readjustment of head position, application of external laryngeal pressure, and assistance by a second person), potential severity of dental trauma (rated as 0 = none, 1 = mild: contact between the blade and the incisors, 2 = moderate: the blade bent the incisors, 3 = severe: the blade bent the incisors and the upper lip), and the laryngeal view using the Cormack-Lehane-score.

#### Subjective assessment

Following the procedure, each participant was tasked with scoring the view of the vocal cords, the handling, the stability of the device, the force applied during intubation, the influence of the mechanical chest compressions, and the overall difficulty of tracheal intubation for each device. To facilitate this, we employed a numeric rating scale ranging from 0 ("excellent/very easy") to 10 ("very poor/very difficult"). Furthermore, participants were asked to rate the subjective requirements using the NASA Task Load Index for each intubation tool. They were asked to rate mental demand, physical demand, temporal demand, performance, effort and frustration on a scale from 0 ("low") to 10 ("high"). Upon completion of all three intubations, participants were instructed to rank the intubation devices based on their preference, with 1 indicating the most preferred device and 3 indicating the least preferred device.

### Data analysis

Prior to the start of the study, a power analysis was performed using G*Power (version 3.1.9.4, Paul F., 2019, Germany). Anticipating an effect size of 0.5 based on data from a pilot study, and with an α-error of 0.05 and a statistical power of 0.8, the sample size calculation determined a minimum group size of 86 participants. All statistical analyses were performed using SPSS software (IBM® SPSS, Version 28.0, IBM Corp., Armonk, NY, USA). Normal distribution was tested using the Kolmogorov-Smirnov test. The data was not normally distributed. Descriptive statistical analysis was used to examine the characteristics of the study population.

The Friedmann test with post-hoc Wilcoxon signed-rank test with Bonferroni correction for multiple comparisons was used to analyze different time points (’time-to-vocal cords’, ’time-to-intubate’, ’time-to-ventilate’), number of intubation attempts, optimization maneuvers, severity of dental trauma, and subjective ratings. Group differences were assessed using the Mann-Whitney U test and the Chi-Quadrat test. Results are presented as median and interquartile range (IQR), with statistical significance set at p<0.05.

## Results

In this randomized crossover trial, we successfully enrolled a total of 90 anesthesiologists. Based on their level of experience, we categorized the participants into two sub-groups for further statistical analysis: the ’limited experience’ sub-group comprised 50 residents with less than six years of clinical experience, while the ’extensive experience’ sub-group included 40 specialists and residents with six or more years of experience. Table 1 provides detailed characteristics of the study population, including exact gender distribution, age, years of clinical experience, total number of intubations performed, VL intubations, and participation in prehospital emergency services. There was no significant difference in gender distribution (p = 0.114).

**Table 1:** Study population characteristics.

	Total study population (N = 90)	Anesthesiologists with limited experience (N = 50)	Anesthesiologists with extensive experience (N = 40)
Gender *			
Female Male	28/90 (31.1%)	19/50 (38.0%)	9/40 (22.5%)
	62/90 (68.9%)	31/50 (62.0%)	31/40 (77.5%)
Age (y) ^$^	34.0 (30,8–42.0)	31.0 (28.8–33.0)	43.0 (39.0–48.5)
Clinical experience (y) ^$^	4.7 (2.2–13.0)	2.5 (1.5–3.5)	14.8 (7.6–19.0)
Emergency medicine expertise (n)	44/90 (48.9%)	14/50 (28.0%)	32/40 (80.0%)
Total number of videolaryngoscopic intubations ^$^	28 (16–40)	20 (14–33.0)	39 (21–54)
Total number of intubations ^$^	946 (518–2020)	575 (379–847)	2141 (1315–3690)

The data are presented as median (inter-quartile range, IQR) or as fraction n/N (%).

* no statistically significant difference in gender distribution (p = 0.114).

^$^ statistically significant difference between the groups (p<0.001).

### Intubation times

The order of intubation devices was randomized for each participant. Each blade was used equally often in positions 1, 2 and 3. The overall intubation success rate was high (CON 100%, MAC 100%, MX 99%). There was one failed intubation attempt due to prolonged intubation time (>120sec) in the MX group. There was a device-dependent result regarding intubation times. The MAC and MX showed a significantly faster ’time-to-vocal-cords’ compared to the CON (p<0.001) with no significant difference between the MAC and MX (p = 0.074). The primary endpoints of ’time-to-intubate’ and ’time-to-ventilate’ were achieved significantly faster with the MAC compared to the CON (’time-to-intubate’ p = 0.01, ’time-to-ventilate’ p = 0.042). Comparing the MAC to the MX, the MAC was timely advantageous (’time-to-intubate’ p<0.001, ’time-to-ventilate’ p = 0.001). There was no significant difference in both intubation times between MX and CON.

The use of the VL with either blade resulted in not only a faster but also a better view of the vocal cords as assessed by the Cormack-Lehane score (MAC and MX vs. CON, p<0.001). There was no difference between MAC and MX (p = 1.0).

Regarding dental trauma, MAC and MX showed significantly less potential trauma in comparison to the CON (p<0.001). On the other hand, we could not find a significant difference for ‘stylet deformation’, ‘optimization maneuvers’ and number of intubation attempts.

There was a significant difference in interruption of the mechanical chest compressions. When using the conventional Macintosh laryngoscope, 5 participants had to interrupt mechanical chest compressions briefly for successful intubation. The median duration of the pause was 8.8s. No interruption was required when using the VL with either blade. (MAC vs. CON p = 0.008, MX vs. CON p = 0.008).

**Fig 2:**
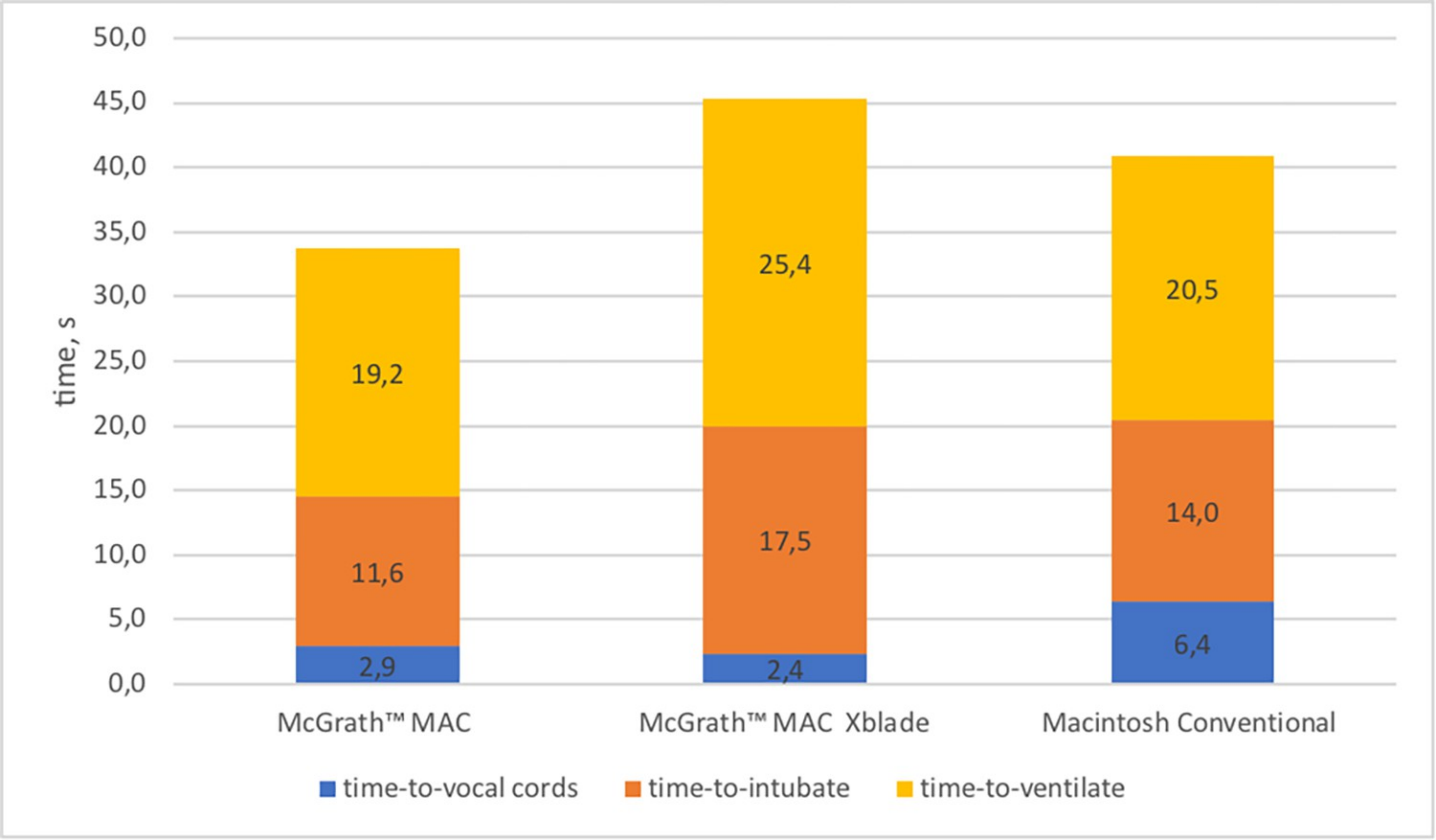
Intubation times for each blade. The ‚time-to-vocal cords‘, ‘time-to-intubate’ and ‘time-to-ventilate’ are displayed for each blade. Times in s.

For detailed results of the analysis please be referred to Table 2 and Fig 2.

**Table 2:** Detailed analysis of the participants results.

	Conventional Macintosh (CON)	McGrath™ MAC (MAC)	McGrath™ MAC XBlade (MX)
Overall success rate, n/N (%)	90/90 (100.0%)	90/90 (100.0%)	89/90 (98.9%)
Esophageal intubation, n/N (%)	0/90 (0%)	0/90 (0%)	0/90 (0%)
Prolonged intubation (>120s), n/N (%)	0/90 (0%)	0/90 (0%)	1/90 (1.1%)
Time-to-vocal-cords (s), median (IQR)	6.4*^,$^ (4.6–10.6)	2.9* (2.0–3.9)	2.4^$^ (1.8–3.3)
Time-to-intubate (s), median (IQR)	14.0 ^$ $^ (10.5–20.4)	11.6 ^$ $^,** (8.5–15.9)	17.5** (11.3–28.1)
Time-to-ventilate (s), median (IQR)	20.5^$ $^ (16.4–26.6)	19.2 ^$ $, §^ (14.5–22.8)	25.4 ^§^ (17.3–38.1)
Number of intubation attempts, n/N (%)			
1	88/90 (97.8%)	90/90 (100%)	86/90 (95.6%)
2	2/90 (2.2%)	0/90 (0%)	4/90 (4.4%)
3 or more	0/90 (0%)	0/90 (0%)	0/90 (0%)
Median	1.0	1.0	1.0
Severity of dental trauma, n/N (%)			
None	7/90 (7.8%)	45/90 (50.0%)	41/90 (45.6%)
Mild	27/90 (30.0%)	41/90 (45.6%)	42/90 (46.7%)
Severe	56/90 (62.2%)	4/90 (4.4%)	7/90 (7.8%)
Median	2.0 *^, $^ (severe)	1 *(mild)	1.0 ^$^ (mild)
Number of optimization maneuvers, n/N (%)			
0	75/90 (83.3%)	86/90 (95.6%)	82/90 (91.1%)
1	14/90 (15.6%)	4/90 (4.4%)	7/90 (7.8%)
2 or more	1/90 (1.1%)	0/90 (0%)	1/90 (1.1%)
Median	0.0	0.0	0.0
Stylet Deformation, n/N (%)			
0	82/90 (91.1%)	84/90 (93.3%)	81/90 (90.0%)
1	7/90 (7.8%)	5/90 (5.6%)	8/90 (8.9%)
2	1/90 (1.1%)	1/90 (1.1%)	1/90 (1.1%)
Median	0.0	0.0	0.0
Interruption of chest compressions (%) (median time, s)	5/90 ^$ $,§§^ (5.6%) (8.8s)	0/90 ^$ $^ (0%)	0/90 ^§§^ (0%)
View, median (IQR)	4.0 *^, $^ (2.0–6.0)	1.0 *(0.0–1.5)	0.8 ^$^ (0.0–1.0)
Handling, median (IQR)	2.0 *^, §§^ (1.0–4.3)	1.0 * (0.0–2.0)	1.0 ^§§^ (0.5–2.5)
Force applied during intubation attempt, median (IQR)	5.0 *^, $^ (2.9–7.0)	1.0 * (0.5–2.0)	1.0 ^$^ (0.5–2.0)
Stability, median (IQR)	2.0 ^$ $^ (1.0–3.0)	1.0 ^$ $^ (1.0–2.0)	1.5 (1.0–2.6)
Handicap due to mechanical chest compressions (IQR)	3.0 *^, $^ (1.0–6.0)	1.0 * (0.5–2.0)	1.0 ^$^ (0.5–2.0)
Overall difficulty of intubation, median (IQR)	4.0 *^, $^ (2.0–6.0)	1.0 * (0.5–2.0)	2.0 ^$^ (1.0–4.0)
Cormack-Lehane Score, n/N (%)			
1	28/90 (31.1%)	80/90 (88.9%)	83/90 (92.2%)
2	54/90 (60.0%)	10/90 (11.1%)	7/90 (7.8%)
3	8/90 (8.9%)	0/90 (0%)	0/90 (0%)
4	0/90 (0%)	0/90 (0%)	0/90 (0%)
Median	2.0 *^, $^	1.0 *	1.0 ^$^

Data are presented as median (inter-quartile range, IQR), number n (%) or as fraction n/N (%). Subjective findings are presented as numeric rating scale (0 to 10, from excellent/very easy to poor/very difficult).

* p<0.001 MAC vs. CON ** p<0.001 MAC vs. MX.

^$^ p<0.001 MX vs. CON ^$ $^ p<0.05 MAC vs. CON.

^§^ p<0.05 MAC vs. MX ^§§^ p<0.05 MX vs. CON.

The subgroup analysis for limited versus extensive experience revealed the following results: Differences between intubation attempts (p = 0.110), intubation success (p = 0.368) and interruption of mechanical chest compressions (p = 0.156) were not statistically significant. However, when using the conventional Macintosh blade participants with extensive experience achieved a significantly faster ‘time-to-vocal-cords’ (p = 0.044), ‘time-to-intubate’ (p = 0.038) and ‘time-to-ventilate’ (p = 0.024) as the participants with limited experience. When using VL (MAC and MX), the participants with limited experience have made up for this deficit and there is no longer a significant difference. There is no significant difference in ‘dental trauma’, ‘stylet deformation’, ‘optimization manoeuvres’ for either blade between the groups. Table 3 displays the detailed subgroup analysis.

**Table 3:** Subgroup analysis, limited experience vs. extensive experience. The data are presented as median (inter-quartile range, IQR) or as fraction n/N (%).

	Anesthesiologists with limited experience (N = 50)	Anesthesiologists with extensive experience (N = 40)
Conventional Macintosh (CON)		
Time-to-vocal-cords* Time-to-intubate* Time-to-ventilate* Maneuvers for optimization Dental trauma Deformation of the stylet Failed Intubation rate, n/N (%)	7.5 (5.3–11.8)	5.8 (4.4–8.0)
	15.3 (11.4–21.5)	12.3 (9.8–18.1)
	21.9 (18.4–29.8)	19.1 (15.8–23.2)
	0 (0–0)	0 (0–0)
	2 (1.0–2.0)	2 (1.0–2.0)
	0 (0–0)	0 (0–0)
	0/50 (0%)	0/40 (0%)
Interruption chest compressions (%, median time s)	4/50 (8.0%, 9.8s)	1/40 (2.5%, 4.3s)
McGrath™ Macintosh (MAC)		
Time-to-vocal-cords Time-to-intubate Time-to-ventilate Maneuvers for optimization Dental trauma Deformation of the stylet Failed Intubation rate, n/N (%) Interruption chest compressions (%, median time s)	3.2 (2.1–4.1)	2.6 (2.0–3.6)
	12.5 (9.3–15.7)	10.4 (8.1–16.3)
	20.0 (15.2–23.7)	16.5 (14.2–22.7)
	0 (0–0)	0 (0–0)
	0 (0–1)	1 (0–1)
	0 (0–0)	0 (0–0)
	0/50 (0%)	0/40 (0%)
	0/50 (0%)	0/40 (0%)
McGrath™ MAC Xblade (MX)		
Time-to-vocal-cords Time-to-intubate Time-to-ventilate Maneuvers for optimization dental trauma Deformation of the stylet Failed Intubation rate, n/N (%) Interruption chest compressions (%, median time s)	2.4 (1.8–3.9)	2.3 (1.7–2.9)
	18.7 (12.5–27.2)	15.5 (9.7–30.4)
	27.1 (19.9–38.7)	23.6 (16.8–37.6)
	0 (0–0)	0 (0–0)
	1 (0–1)	1 (0–0)
	0 (0–0)	0 (0–1)
	1/50 (2.0%)	0/40 (0%)
	0/50 (0%)	0/40 (0%)

* statistically significant difference between the groups (p< 0.05).

### Subjective evaluation

All 90 participants completed the questionnaire completely and correctly, so 90 questionnaires could be included in the analysis. The detailed results of the analysis are shown in Table 4. The view of the vocal cords using a video-based intubation tool was rated significantly better than using a conventional tool (MAC or MX vs. CON, p<0.001). There was no significance between MAC and MX (p = 0.791). Regarding the handling of the intubation device and the force required during intubation, MAC and MX were rated better than CON (handling: MAC vs. CON p<0.001, MX vs. CON p = 0.01; force used during intubation: MAC or MX vs. CON p<0.001). Regarding the stability of the intubation device, the MAC was rated superior to the CON (p = 0.027).

**Table 4:** Subjective workload assessment using the NASA Task-Load Index.

	Conventional Macintosh (CON)	McGrath™ MAC (MAC)	McGrath™ MAC Xblade (MX)
Mental demand	2.5* ^§§^ (1.3–5.0)	2.0 *(1.0–2.6)	2.0 ^§§^ (1.0–3.0)
Physical demand	4.0 * ^$^ (3.0–6.0)	2.0 *(1.0–3.0)	2.0 ^$^ (1.0–3.0)
Temporal demand	4.0 *(2.5–6.3)	2.0 * ^§^ (1.0–4.1)	3.0 ^§^ (1.5–6.0)
Performance	2.0 ^$ $^ (1.3–4.0)	1.8 ^$ $^ (1.0–2.5)	2.0 (1.0–4.0)
Effort	4.0 * ^$^ (2.5–6.0)	2.0 * (1.0–3.0)	2.0 ^$^ (1.0–4.0)
Frustration	2.0 ^$ $^ (1.0–3.5)	1.0 ^$ $^ (0.5–2.5)	2.0 (1.0–4.0)

Data are presented as median (inter-quartile range, IQR). Subjective findings are presented as numeric rating scale (0 to 10, from low to high).

* p<0.001 MAC vs. CON ** p<0.001 MAC vs. MX.

^$^ p<0.001 MX vs. CON ^$ $^ p<0.05 MAC vs. CON.

^§^ p<0.05 MAC vs. MX ^§§^ p<0.05 MX vs. CON.

The negative influence of chest compressions was rated significantly higher for the CON than for the MAC (p<0.001) or the MX (p<0.001). Participants were asked to rate the overall difficulty of intubation with each device. While the overall difficulty of intubation was rated statistically equal for the MAC and the MX (p = 0.121), intubation with the CON was rated more difficult than using the MAC or the MX. (both p<0.001).

**Fig 3:**
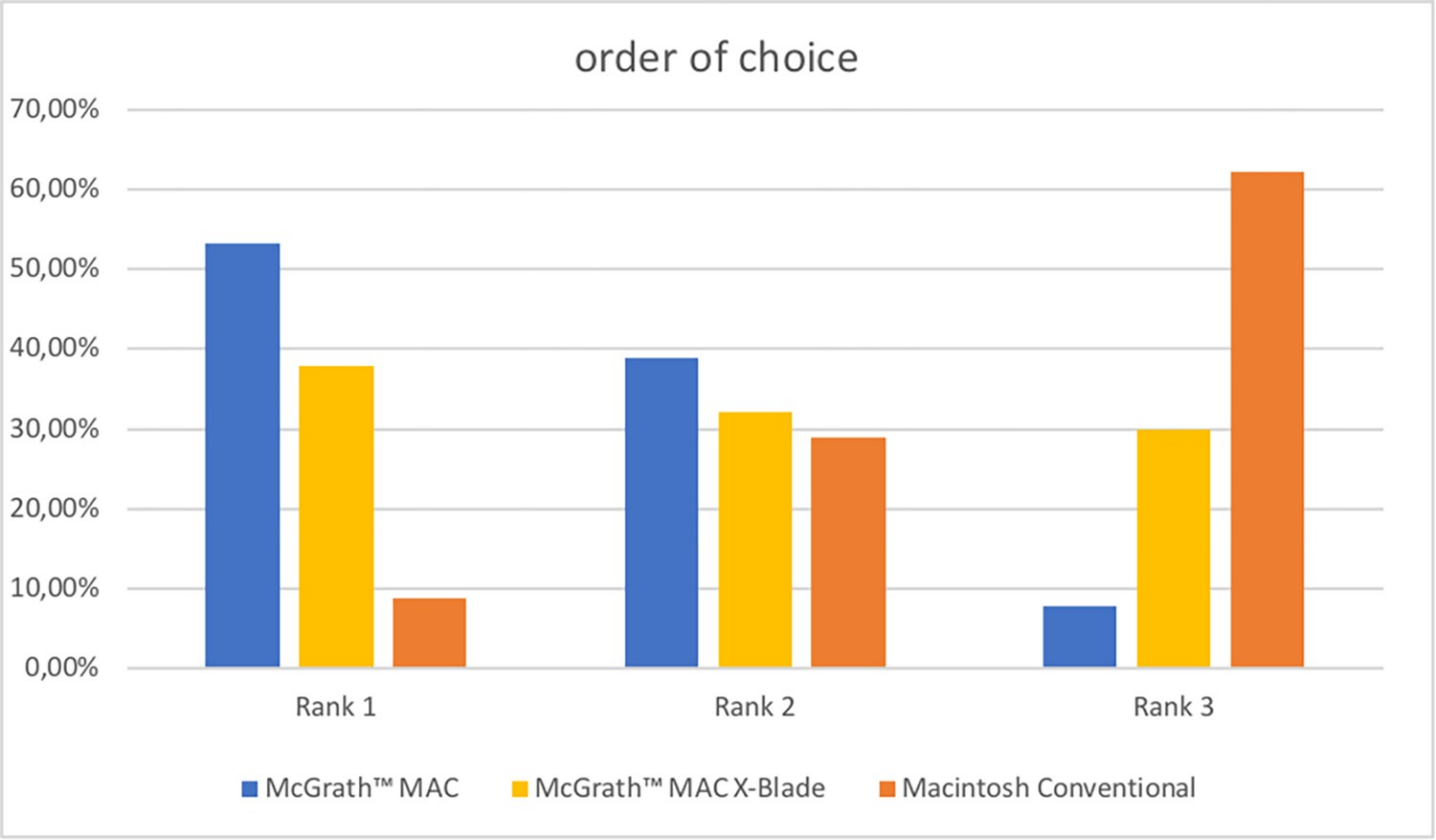
Subjective results. The participants were asked to bring the devices in an order of their preferences with rank 1 the most preferred, rank 3 the least preferred device.

At the end of the questionnaire, clinicians were asked to rank the devices in order of preference. Most clinicians (48/90, 53.3%) chose the MAC as their preferred device and the CON was chosen as the least preferred device by most clinicians (56/90, 62.2%). See Fig 3 for detailed results.

### Workload assessment

To assess the perceived workload during intubation under mechanical chest compressions, all participants were asked to complete the NASA Task-Load-Index. In one case the index only for the CON was not filled in, the rest was complete. We found significant differences between the conventional and both VL blades: Mental and physical demands were rated lower for the MAC and the MX compared to the CON (mental: MAC vs. CON p<0.001, MX vs. CON p = 0.013; physical: MAC or MX vs. CON p<0.001). Temporal demands were subjectively higher with the CON and the MX than with the MAC (MAC vs. CON p<0.001, MAC vs. MX p = 0.019). The MX and the CON were rated statistically equivalent (p = 0.089). Satisfaction with one’s own performance was rated highest for the MAC (MAC: 1.8, MX: 2.0, CON: 2.0). There was a statistically significant difference compared to the CON (p = 0.002). Personal effort was highest with the CON (CON 4.0, MAC 2.0, MX 2.0, CON vs. MAC or MX p<0.001). The rate of frustration was also rated highest for the CON and the MX (CON 2.0, MX 2.0, MAX 1.8, CON vs. MAX p = 0.004). Table 4 displays the detailed results.

## Discussion

ETI during cardiopulmonary resuscitation presents unique challenges that differ markedly from those encountered in controlled settings such as the operating room. These challenges are magnified in the prehospital environment where factors such as adverse environmental conditions, suboptimal patient positioning, and varying levels of provider expertise contribute to increased difficulty. The McGrath™ videolaryngoscope is widely used in prehospital emergency medical services due to its compact and portable design. A direct comparison of the Macintosh-like blade MAC, the hyperangulated blade MX and a conventional Macintosh blade under resuscitation conditions is lacking. This prospective, randomized study among 90 anesthesiologists aimed to evaluate the efficacy and utility of the McGrath™ videolaryngoscope, specifically comparing the Macintosh-like MAC blade, the hyperangulated MAC Xblade and a conventional Macintosh blade under mechanical chest compressions.

Our study demonstrated a high overall success rate for intubation, ranging device dependent from 99–100%. This aligns with previous studies that have reported similarly high success rates [15] using the MAC blade in simulated difficult airway scenarios [16].

The analysis of the intubation times highlights notable differences between the blade types. Specifically, both MAC and MX blades showed faster times to visualize the vocal cords compared to the conventional blade.

The primary endpoints of our study—’time-to-intubate’ and ’time-to-ventilate’—revealed different results. The MAC blade proved superior in terms of ‘time-to-intubate’ and ‘time-to-ventilate’ not only against the conventional blade but also against the video-based MX blade. The advantage of the hyperangulated blade over the Macintosh in the ‘time-to-vocal cords’ is not only equalized, but the CON is even faster in ‘time-to-intubate’ and ‘time-to-ventilate’. However, these differences proofed no statistical significance (Fig 2). Overall, the time differences between the individual blades are small. The extent to which a time difference of a few seconds is clinically relevant to patients’ outcome cannot be answered at this time. In a multicenter study, Prekker et al. [9] also showed a time difference of approximately 8 seconds between videolaryngoscopy and direct laryngoscopy. In addition to a significantly better first-pass effect of videolaryngoscopy, no clear conclusions could be drawn regarding secondary outcomes such as hypoxia and hypotension [9]. We observed one failed intubation due to a prolonged intubation time of >120 seconds with the hyper-angulated MX blade. This suggests that while the hyperangulated MX blade provides excellent visualization, it may present challenges during the actual tube placement. This phenomenon described by Klein-Brueggeney as ’you see that you fail’ [17] is consistent with other studies that have observed difficulties in tube placement despite clear visualization with hyperangulated blades in pediatric and adult settings [11,17]. This might be aggravated under the stress of ongoing chest compressions.

The potential dental injuries were reduced with both the MAC and MX blades compared to conventional laryngoscopes. Our results are consistent with studies on difficult intubations, which also found lower incidences of dental trauma with VL [11,18]. However, a Cochrane review by Hansel et al. provided mixed results regarding the comparison of dental injuries between VL and conventional laryngoscopes, highlighting the need for further research to solidify these findings [19].

High-quality, continuous chest compressions with few or no interruptions are critical to the quality of CPR. Only when using the conventional Macintosh blade did five users have to ask for a brief interruption of chest compressions. This was not necessary when using the VL. In four cases these were less experienced users, one case came from the more experienced group. The participants rated the negative influence of chest compressions significantly higher when using a conventional laryngoscope over the two VL-blades. Our findings indicate that the experience level of the operator might impacts intubation times with conventional laryngoscopes during CPR. The group with extensive experience was significantly faster while using the CON compared to the less experienced group. This effect is mitigated with the use of VL. This observation aligns with the work of van Schuppen, who demonstrated that VL could expedite and secure intubation for less experienced practitioners [8]. Additionally, Griesdale’s review, which included both experienced and inexperienced clinicians, noted a diminished disparity in intubation efficiency when using VL [20]. Our study supports these findings, showing that both the MAC and MX blades enabled quicker intubation with fewer interruptions to chest compressions compared to the CON, regardless of the operator’s experience level. This finding is significant for prehospital and emergency settings where provider experience can vary widely, and rapid, successful intubation is critical.

Airway management during CPR is inherently stressful and can exacerbate the already high levels of stress experienced by the resuscitation team. There is evidence suggesting that self-perceived stress is a predictor for delayed initiation and prolonged pauses in chest compressions among medical residents [21]. Our study revealed that the use of VL significantly reduced the self-perceived mental and physical demands of securing the airway. Participants reported lower levels of personal effort required for successful intubation during ongoing chest compressions when using VL. Notably, the Macintosh-like VL blade MAC was rated superior to the hyperangulated MX blade in terms of perceived time pressure.

Reducing stress and improving the efficiency of airway management can potentially enhance overall resuscitation outcomes. The objective and subjective results of our study suggest that VL appears to be a superior choice over conventional laryngoscopy during mechanical chest compressions, especially for less experienced users. Macintosh-like VL blades, such as the McGrath™ MAC, appear to be even preferable to hyperangulated blades in terms of time to intubation and ventilation, as well as in aspects of perceived workload during intubation.

## Limitations of the study

While our study provides valuable insights, it is important to recognize its limitations. Firstly, as a manikin-based study, it cannot fully replicate the complexities and variables present in real-life clinical scenarios. Thus, the results should be interpreted with caution and validated through clinical trials. Secondly, there is evidence suggesting that the positive effects of hyperangulated blades observed in manikin studies may be underestimated and could be more pronounced in actual clinical settings [8]. The time difference between each blade is small. The extent to which this may be clinically relevant cannot be answered by this study. Further research involving larger sample sizes and clinical environments is necessary to corroborate our findings. In order to create the most consistent conditions possible for all participants, we used a device for mechanical chest compressions. The extent to which these intubation conditions are transferable to cases in which manual chest compressions are performed is uncertain.

Additionally, the study design inherently lacks blinding, which could introduce bias. Despite randomizing the sequence of blade use, a potential training effect cannot be entirely ruled out. This factor might have influenced the results, as participants could have become more proficient with each subsequent intubation attempt. The evaluation of the intubation figures shows a limited number of videolaryngoscopies performed, especially in the group with limited experience. The extent to which this has affected the handling of the videolaryngoscope, in particular the hyperangulated blade is unclear.

## Conclusion

In conclusion, our study contributes to the growing body of evidence supporting the use of VL in airway management during CPR. The McGrath™ videolaryngoscope using the McGrath™ MAC blade, demonstrates significant advantages in terms of intubation time, user-friendliness, and reduced stress levels among operators. While the hyperangulated MX blade provides excellent visualization, its practical challenges during tube placement warrant consideration. Further clinical studies are essential to validate these findings and establish more definitive guidelines for the use of VL in resuscitation settings. Through continued research and innovation, we hope to contribute to improving resuscitation techniques and ultimately enhance patient survival and recovery outcomes.

## Supporting information

S1 Dataset This is the S1 Dataset for data analysis. (SAV)
